# Methods of a large prospective, randomised, open-label, blinded end-point study comparing morning versus evening dosing in hypertensive patients: the Treatment In Morning versus Evening (TIME) study

**DOI:** 10.1136/bmjopen-2015-010313

**Published:** 2016-02-09

**Authors:** David A Rorie, Amy Rogers, Isla S Mackenzie, Ian Ford, David J Webb, Bryan Willams, Morris Brown, Neil Poulter, Evelyn Findlay, Wendy Saywood, Thomas M MacDonald

**Affiliations:** 1 University of Dundee, Ninewells Hospital and Medical School, Dundee, UK; 2 Robertson Centre for Biostatistics, University of Glasgow, Glasgow, UK; 3 Centre for Cardiovascular Science, University of Edinburgh, Edinburgh, UK; 4 NIHR UCL Hospitals Biomedical Research Centre, Institute of Cardiovascular Science, UCL, London, UK; 5 University of Cambridge, Cambridge, UK; 6 Imperial College London, London, UK

**Keywords:** CLINICAL PHARMACOLOGY, EPIDEMIOLOGY

## Abstract

**Introduction:**

Nocturnal blood pressure (BP) appears to be a better predictor of cardiovascular outcome than daytime BP. The BP lowering effects of most antihypertensive therapies are often greater in the first 12 h compared to the next 12 h. The Treatment In Morning versus Evening (TIME) study aims to establish whether evening dosing is more cardioprotective than morning dosing.

**Methods and analysis:**

The TIME study uses the prospective, randomised, open-label, blinded end-point (PROBE) design. TIME recruits participants by advertising in the community, from primary and secondary care, and from databases of consented patients in the UK. Participants must be aged over 18 years, prescribed at least one antihypertensive drug taken once a day, and have a valid email address. After the participants have self-enrolled and consented on the secure TIME website (http://www.timestudy.co.uk) they are randomised to take their antihypertensive medication in the morning or the evening. Participant follow-ups are conducted after 1 month and then every 3 months by automated email. The trial is expected to run for 5 years, randomising 10 269 participants, with average participant follow-up being 4 years. The primary end point is hospitalisation for the composite end point of non-fatal myocardial infarction (MI), non-fatal stroke (cerebrovascular accident; CVA) or any vascular death determined by record-linkage. Secondary end points are: each component of the primary end point, hospitalisation for non-fatal stroke, hospitalisation for non-fatal MI, cardiovascular death, all-cause mortality, hospitalisation or death from congestive heart failure. The primary outcome will be a comparison of time to first event comparing morning versus evening dosing using an intention-to-treat analysis. The sample size is calculated for a two-sided test to detect 20% superiority at 80% power.

**Ethics and dissemination:**

TIME has ethical approval in the UK, and results will be published in a peer-reviewed journal.

**Trial registration number:**

UKCRN17071; Pre-results.

**Strengths and limitations of this study**

- Use of technology including an electronic case report form and record linkage to identify potential end points allows efficient data management.
- The open-label design provides good external validity. The end-point committee is blinded to treatment randomisation.
- Limitations include the generalisability of results based on participants who have an email address and are technologically literate.

## Introduction

Nocturnal blood pressure (BP) has consistently been found to be a better predictor of cardiovascular outcome than daytime BP. As the day:night BP ratio increases, cardiovascular risk appears to decrease.^1–4^ There is also evidence to suggest that antihypertensive drugs taken in the evening rather than in the morning reduce nocturnal blood pressure to a greater extent,^5–7^ and might have more benefit in hypertension alone^8^ ^9^ hypertension with renal disease^10^ ^11^ and hypertension with diabetes.^12^ A recent study that examined the effect on nocturnal blood pressure of drugs taken in the evening in participants with resistant hypertension found that bedtime dosing resulted in significantly lower 24 h means of systolic and diastolic BP (by 4.1/1.5 mm Hg) and that this difference between groups was driven by asleep BP (9.7/4.4 mm Hg lower).^13^ The most recent and most compelling evidence in favour of nocturnal dosing comes from the Monitorización Ambulatoria para Predicción de Eventos Cardiovasculares (MAPEC) study which randomly assigned 2156 hypertensive patients to take all of their antihypertensive drugs on awakening or to take one or more of them at bedtime.^14^ ^15^ All patients in this study underwent ambulatory blood pressure monitoring (ABPM) at baseline and yearly, and all wore wrist activity metres to control for the effects of activity on BP and to determine when participants were asleep.^16^ Participants in the study who took one or more antihypertensive drugs at bed-time had 68 cardiovascular events whereas those who took all medication on awakening had 187 events; a relative risk reduction of 64% (p<0.001). Forty-eight-hour ABPM showed lower systolic BP (122.1 mm Hg morning dosing vs 120.8 mm Hg evening dosing p=0.029) with evening dosing and this difference was due to lower asleep systolic blood pressure (116.1 mm Hg morning dosing vs 110.9 mm Hg evening dosing, p<0.001). The MAPEC study was limited in that it was not prospectively powered, the process of randomisation was not reported, and the end points included some unusual cardiovascular events, and were not independently adjudicated.

Further studies are needed to establish the optimal time of dosing for hypertensive patients.^17^ ^18^ Therefore, the present study aims to substantiate the findings of the MAPEC study and test the hypothesis that nocturnal dosing of antihypertensive medication reduces cardiovascular events compared with conventional morning dosing. Secondary questions will examine whether there are any downsides to nocturnal dosing and if nocturnal dosing is acceptable to patients, for example, whether nocturnal dosing of diuretics causes unacceptable nocturia will be evaluated. Data will also be collected on nocturnal hypotension and its consequences (eg, falls and fractures).

## Methods

### Trial design

**Figure 1:**
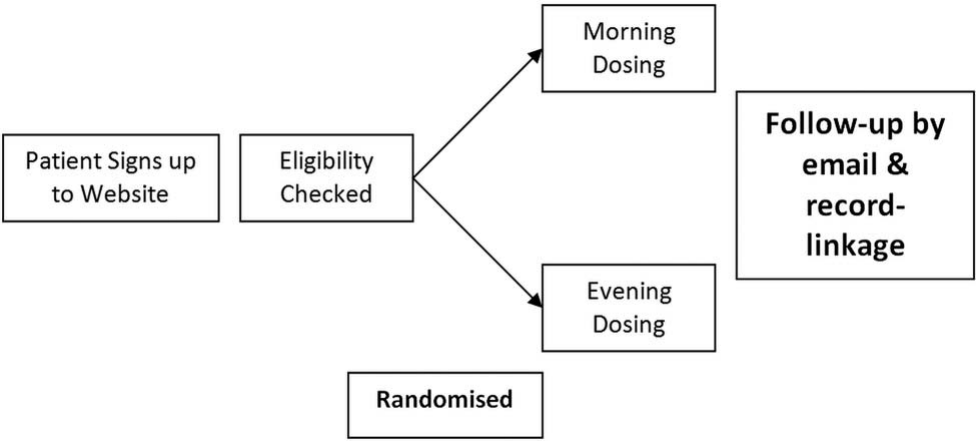
Schematic diagram of study.

**Figure 2:**
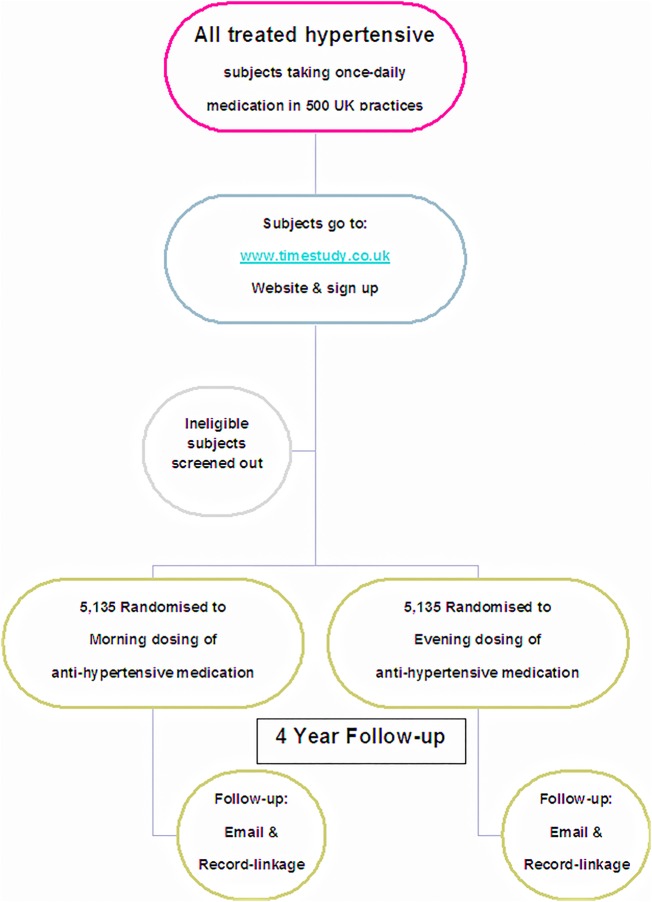
TIME study flow diagram.

The Treatment In Morning versus Evening (TIME) study is a prospective, randomised, open-label, blinded end-point (PROBE) design^19^ controlled clinical trial. TIME (http://www.timestudy.co.uk) builds on a successful novel methodology to track patient outcome using information technology (IT). The project will be conducted on a secure web portal with patients signing up on-line and being followed up by email and record-linkage to national databases to identify hospitalisations and deaths.^20^ ^21^ End points will be detected by record-linkage to hospitalisation, death certification records and regular email contacts with patients and nominated surrogates. Primary or secondary care records and physicians will be contacted for further data, if required (all patients must consent to their medical records and physicians being accessed or contacted). All end-point adjudication will be blinded to dosing time and original medical records will be retrieved for hospitalisation events to validate end points. The schematics and flow of the study can be seen in figures 1 and 2. The TIME study underwent a pilot phase between 2011 and 2014 to determine feasibility of a full study. At that time the study was not adopted by the UKCRN and so trial registration was not possible. There were doubts as to the viability of the study being conducted online and whether there would be sufficient recruitment uptake. Once the study was deemed sustainable, funding was in place, and the clinical trials networks adopted the study, it was prospectively registered in ISCRTN and the pilot transferred to the full study.

### Recruitment strategy

In an effort to recruit the necessary number of participants, the full study will seek to recruit by advertising to hypertensive patients in the UK from primary care, secondary care and from databases of patients who have previously consented to being contacted about potential research projects. Collaborating general practitioners (GPs) will bring this trial to the attention of all treated hypertensive patients in their practices by sending letters advertising the study. Secondary care clinics will also send letters advertising the study to previous clinic attendees and display advertising posters in appropriate patient waiting areas. Databases of consented patients will be utilised where appropriate, such as UK Biobank,^22^ Tayside Bioresource and GoShare,^23^ contacting patients and advertise the study to them. The Database owners will send approved letters on behalf of TIME. The inclusion and exclusion criteria for TIME are listed in boxes 1 and 2. Potential study participants are invited to complete an initial online study registration. This process requires them to confirm their eligibility against the study inclusion and exclusion criteria before allowing them to proceed and complete enrolment.

Box 1 **TIME inclusion criteria**

Inclusion criteria

- Both diagnosed and treated for hypertension (all forms) with at least one antihypertensive drug.
- Age ≥18 years.
- Have a valid email address.

Box 2 **TIME exclusion criteria**

Exclusion criteria

1. Participants who take two times a day antihypertensive therapy.
2. Participants who work shift patterns that include a night shift.
3. Participants who are unwilling to consent to:
  - Follow-up
  - Providing a surrogate to be contacted and/or
  - Their family practice releasing follow-up clinical data
  - Their physical case records abstracted if required
  - Their electronic case records searched and abstracted if required
  - Their consent form to be copied to authorities from whom the study team is requesting medical data.
4. Those participating in another clinical trial or who have done in the past 3 months.

### Intervention

Participants within the study are randomly allocated to one of two dosing periods. Participants allocated to morning dosing are advised to take all of their usual blood pressure lowering medications between 6:00 and 10:00 throughout the study. Those allocated to evening dosing are instructed to take all their blood pressure lowering medications between 20:00 and midnight. There is no other intervention in the study and participants continue to attend their usual GP or outpatient clinic for routine hypertension follow-up.

### Follow-up

Patients will be followed up after 1 month of being assigned a randomised time of dosing, and then every 3 months. Contact will be made by automated email with a reminder email sent 14 days later. If the follow-up has still not been completed then 14 days after the first email reminder has been sent an email will be sent to the participant's surrogate asking them to respond as to the well-being of the participant. As a last resort, telephone calls to patients or primary care physicians can be used to contact participants and to ask them to complete follow-ups. Participants can enter follow-up data at any time during the trial by logging into the secure website and are not restricted to waiting for automated emails. A final record linkage will be performed 3 months after the end of the trial in order to capture late recorded events and a final 3 months will be spent finalising adjudications, locking the database and carrying out the analysis.

### Consenting participants

**Figure 3:**
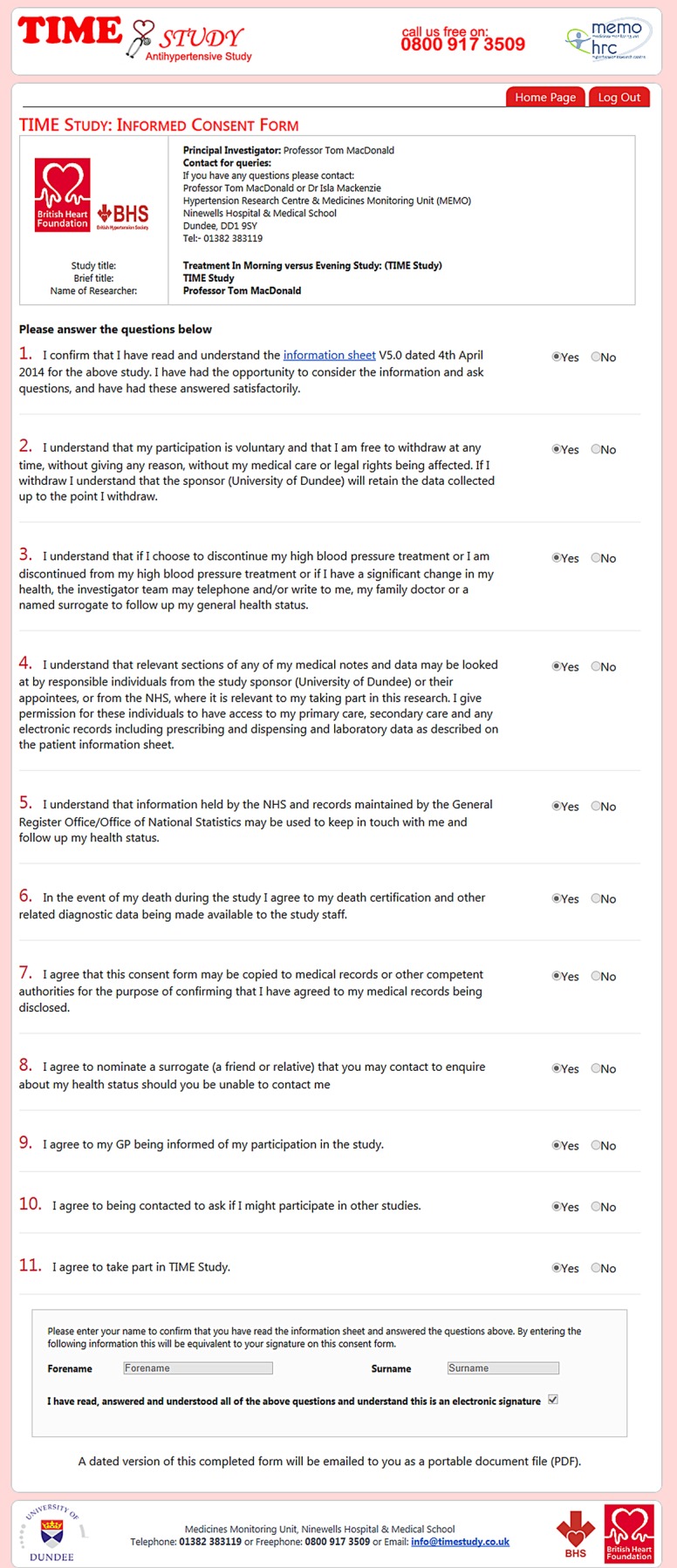
Consent form.

Patients will be required to fill in an electronic consent form (figure 3). The consent form will consist of check boxes to attest to consent for each aspect of the trial. A final check box will be marked ‘I have read, answered and understood all of the above questions and understand this is an electronic signature’. The consent form will record the participant's internet protocol (IP) address along with their email address and a time stamp of when the form was electronically submitted. The consent form will be sent to participants electronically in portable document format (PDF) format for their own records. The decision of a participant to participate in clinical research is voluntary and will be based on a clear understanding of what is involved. A patient information sheet will be available on the TIME website with detailed information about the trial. The consent process will be conducted by participants and entirely via the study website, without the active participation of study personnel. Participants will be given opportunities to clarify any points they do not understand, and to ask for more information by using a ‘Contact Us’ link, or the free phone telephone number, on the study website.

### Withdrawal

Participants will be free to withdraw from the TIME study at any point. A participant can withdraw from:

- Taking medication according to the time of randomisation.
- Receiving further follow-up emails.
- Follow-up of their outcome by record linkage, contacting their surrogate or by contacting their family doctor.

Part of the objective of this study is to determine if subjects can continue to take medication at their randomly allocated time, and indeed, we expect that a proportion of participants will withdraw from taking medication at their allocated time. We shall evaluate the withdrawal rate and record reported reasons for their withdrawal.

## Randomisation

### Computer randomisation

Randomisation is carried out centrally using randomly generated bits (0 and 1 s) which are then allocated to participants (0=morning, 1=evening) sequentially. Randomised status is confirmed by automated email sent to the participant.

### Treatment allocation

Participants are randomised to take their antihypertensive medications in the morning (range 6:00–10:00) or in the evening (range 20:00–midnight). Treatment allocation is not made available to treating physicians unless there is a specific individual clinical need.

### Study population

Hypertensive patients aged 18 or over, in the UK, prescribed one or more once daily antihypertensive drug therapies, and, who have a valid email address (see box 1).

## Trial end points

### Primary end point

The primary end point is first occurrence after randomisation of vascular death or hospitalisation for the composite end point of non-fatal MI or non-fatal stroke, in keeping with major cardiovascular disease (CV) end-point trials.^24^

### Secondary end points

Secondary end points (in rank order) will include:

- Each component of the primary end point:
  Hospitalisation for non-fatal stroke
  Hospitalisation for non-fatal MI
  Cardiovascular death
- All-cause mortality
- Hospitalisation or death from congestive heart failure.

### End-point adjudication

End points will be adjudicated by an independent end-point committee who will be blind to dosage time allocation. Hospitalisation data will be retrieved in collaboration with the medical records departments. Copies of the patient consent forms will be supplied to facilitate this process. The records will be processed, abstracted and suitably redacted to produce anonymised end-point packages for the end-point committee. This committee will have due regard of the published consensus diagnostic criteria for myocardial infarction,^1^ ^25^ stroke,^26^ vascular death, and heart failure.^27^

### Adverse events

**Figure 4:**
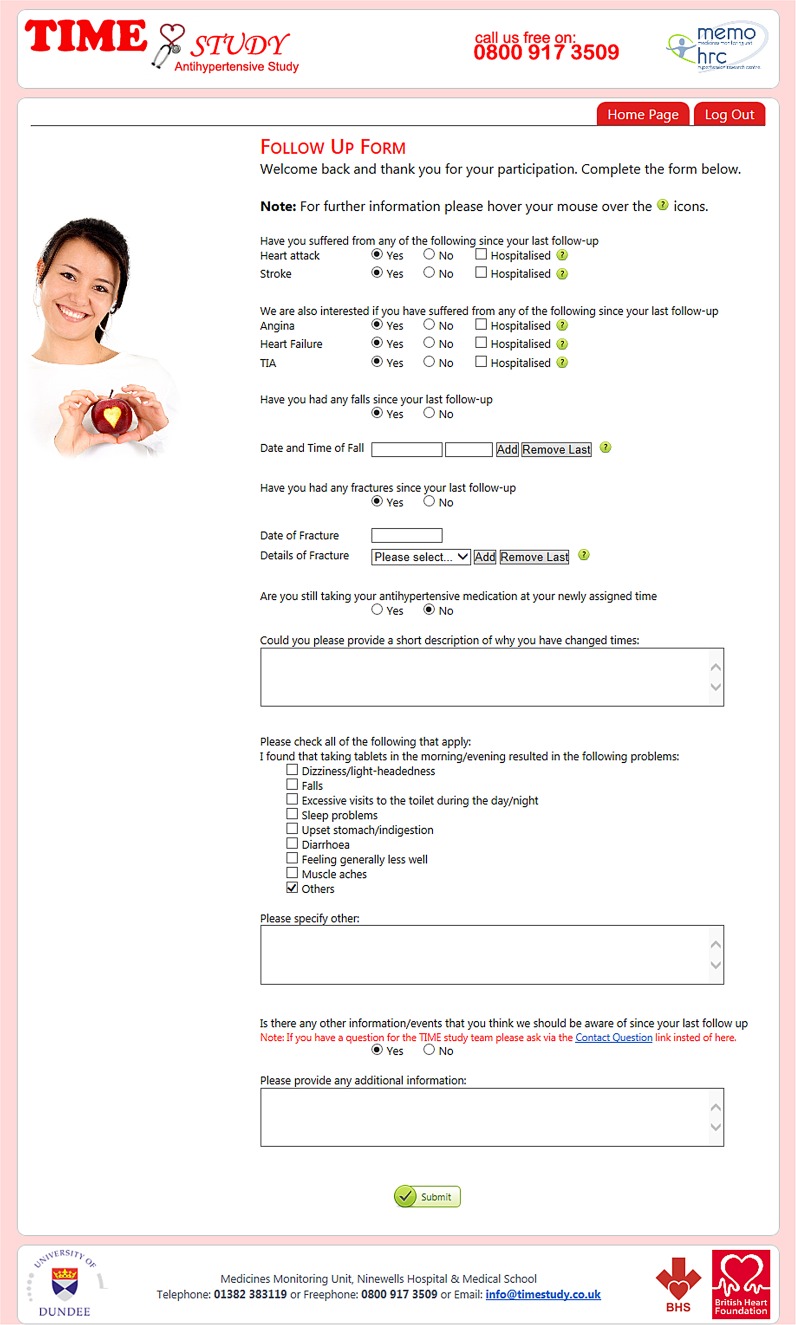
Follow-up form.

**Figure 5:**
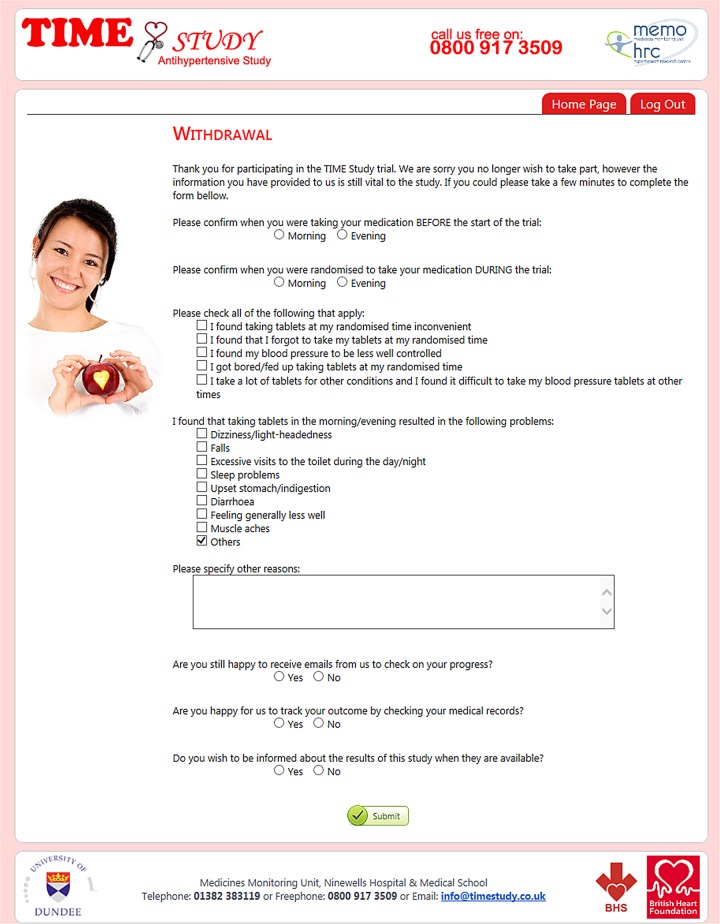
Withdrawal form.

Recording of serious adverse events (AEs) will focus on deaths and hospital admissions as these will be obtained systematically by record linkage. The present study will collect only non-serious AEs associated with changing the time of dosing. Follow-up (figure 4) and withdrawal (figure 5) questionnaires include a mix of open and closed questions regarding medical events and potential AEs. In the case of an AE, patients will judge whether they are happy to continue taking treatment at the time randomised or whether they wish to change or revert to the alternative time of dosing, or, whether they wish to discontinue the study.

An additional reason for carrying out the study is to determine whether participants can tolerate taking medication in the evening. This is prospectively measured. Participants self-report AEs associated with dosing time and whether nocturnal diuretic dosing has been acceptable to them and whether falls and fractures are increased as a potential surrogate for nocturnal hypotensive events.

### Statistics

The primary outcome will be a comparison of time to first event comparing morning versus evening dosing allocation. This outcome will be analysed using a Cox proportional hazards model including treatment group as a covariate. The p value for the treatment effect will be based on the Wald statistic. Point estimates and 95% CIs will be calculated for the treatment effect HR. Time to event curves will be based on cumulative incidence functions. Time to event secondary outcomes will be analysed using similar methods. The proportionality of hazards assumptions will be assessed using Cox models including treatment group by log (time) interactions.

Serious AEs and AEs will be tabulated by treatment group by system organ class and preferred term. All main analyses will be on an intention-to-treat basis.

### Data collection and retention

This study will capture data directly from patients or their nominated surrogates and by record linkage to hospitalisations and deaths. Consent will be gathered from patients to have access to their healthcare records, diagnostic or death data, their acute care summary, their paper or electronic records, their paper or electronic primary care records, and, permission to contact their physicians for further data. Data will be validated at point of entry into the TIME database and at regular intervals during the study. Data will be held securely within the Medicines Monitoring Unit (MEMO) at Ninewells Hospital and Medical School. To enable evaluations and/or audits, the investigators will keep records, including the identity of all participating patients, all original informed consent data, AE data and any source documents. The records will be securely retained and archived by the study sponsor according to ICH, GCP and local regulations. Participating individuals will be able to have sight of their own data on request and will be allowed to comment on perceived inaccuracies therein.

### Data protection

The study will comply with the requirements of the Data Protection Act 1998 with regard to the collection, storage, processing and disclosure of personal information and will uphold the Act's core principles. Access to collated participant data will be restricted to appropriate study staff. Published results will not contain any personal data that could allow identification of individual participants.

### Sample size: evidence of feasibility and power calculation

For a two-sided test to detect a HR of 0.8 for the primary outcome (evening dosing vs morning dosing) at 80% power, 631 events are needed. Based on the anticipated profile of participants, a trial with an average 4-year follow-up period would need to randomise 9780 participants. Since the primary analysis is intention to treat and because few participants are likely to withdraw consent for record-linkage follow-up, only relatively minor (<5%) inflation of the 9780 participants is required to compensate. A target of 10 269 participants will be randomised. Note that falling cardiovascular event rates in the UK may require a revision of the sample size.

## Substudies

### Home BP substudy

Randomised members of the TIME study are asked, during enrolment, if they are prepared to participate in a substudy and submit home BP readings. Participants who own their own home BP monitor are asked to record and submit BP readings at baseline and then regularly throughout the study. All types and brands of home BP monitors are accepted. A comparison of data collected from non-validated versus validated equipment will be conducted. Participants are issued with instructions to take and submit three sets of readings, morning and evening, for 4–7 days. Specific advice is provided about how to take the measurements in accordance with National Institute for Health and Care Excellence guidelines. The results will demonstrate the effect on home BP of morning versus evening dosing and may help validate adherence to nocturnal dosing.

### Cognitive function substudy

Hypertension has been shown to be a predictor of mild cognitive decline.^28^ ^29^ Previous studies have indicated that, particularly at age ≥70 years,^30^ ^31^ BP-lowering has relatively little impact on the rate of cognitive decline; but most trials used morning dosing only. The main purpose of cognitive testing in TIME will be to detect temporal change, particularly any sustained reduction in performance, associated with time of antihypertensive dosing. We propose to use combined telephone testing with the Montreal Cognitive Assessment Test and the Trends in Cognitive Sciences^29^ to assess cognitive function in consenting participants in the TIME trial. Previous experience shows that testing takes between 15 and 30 min and is well tolerated by patients.

### Genetics substudy

All participants will be asked if they are prepared to consent to participate in a genetics substudy where their DNA will be bio-banked for possible future genetic studies. Participants who consent will be asked to provide a cheek swab or saliva collection by post.

## Competing studies

We are not aware of any competing studies that would conflict with the TIME study.

### Early stopping

The Independent Data and Safety Monitoring Committee (IDMC) will review un-blinded study results on a regular basis. Taking a balanced view of all accumulated data, levels of statistical significance and the seriousness of events the IDMC might recommend stopping of the study because of an excess of serious AEs in the evening dosing group. The IDMC will also have the opportunity to make a recommendation of early stopping because of overwhelming evidence of benefit from evening dosing based on interim analyses after approximately 50% and 75% of the target number of adjudicated study outcomes have been observed. Overwhelming evidence of benefit is defined as evidence of benefit of evening dosing versus morning dosing (p<0.001).

## Ethics and dissemination

### Steering committee and IDMC

The TIME steering committee oversees the appropriate scientific and ethical conduct of the trial, provides advice to the Study Sponsor, advises on the conduct and analysis of the study, and approves all publications and substudies. The Committee will operate through meetings, teleconferences and emailings. The Steering Committee will be made up of invited experts, the Chief Investigator, the chair of the end-point Committee plus the coapplicants. The Steering Committee will meet at least annually.

The IDMC is completely independent and comprises experts in the field including clinicians with experience in hypertension and an expert trial statistician. The committee receives un-blinded data and has the power to recommend modifications to the conduct of the study, including early discontinuation based on a risk/benefit assessment of the study data. It will meet at least annually and report to the Steering Committee.

### Sponsorship: monitoring, audit, quality control and quality assurance

The study sponsor is the University of Dundee, who are responsible for monitoring and quality assurance. MEMO has conducted independent penetration testing of the TIME website and the Tayside Medical Science Centre (TASC) is assisting MEMO in ensuring quality control for the study. The pilot study was funded by the British Hypertension Society; the full trial is funded by the British Heart Foundation.

### Protocol amendments

Changes in research activity, except those necessary to remove an apparent, immediate hazard, will be reviewed and approved by the Chief Investigator and Sponsor. Amendments to the protocol will be submitted in writing for approval by the appropriate regulatory and ethical authorities prior to implementation.

### Collaborating investigators

Collaborating investigators will be responsible for dealing with the local issues of bringing the trial to the attention of possible participants either in clinics or in primary care. Since all patients by their own volition decide to go to the study website and sign up, the usual investigator/study subject relationship is not present.

### Confidentiality

All data will be held securely with restricted access. Clinical information will not be released without the written permission of the participant, except as necessary for auditing by the sponsor, its designee, regulatory authorities or the research ethics committee.

### Trial registration

TIME is registered as ISRCTN: 18157641 and with a UKCRN ID: 17071. The trial is performed in line with Good Clinical Practice guidelines and International Society of Pharmacoepidemiology (ISPE) Good Pharmacoepidemiology Practice Guidance.^28^

### Dissemination

The results of the trial will be published in a peer-reviewed scientific journal and made available to participants.

## Discussion

The trial design of TIME allows a large study to be undertaken efficiently and cost-effectively by maximising the benefits of modern technology, including use of an electronic case report form and following up patients using record linkage. All data are entered directly by participants, thereby helping to reduce researcher time and costs. Participant's GPs are made aware of their participation in the study but there is no significant workload implication and GPs are not required to submit data. The study allows participants to self-enrol, consent and provide data in a secure online environment. The TIME study has been awarded a grant from the BHF to write to 300 000 patients across the UK to achieve its target of randomising over 10 000 participants.

If the TIME study shows definite benefits of dosing antihypertensive medication in the evening rather than the morning, this would represent the most cost-effective advance in the treatment of hypertension and the prevention of CV disease in recent years. TIME will also establish whether participants can tolerate nocturnal dosing and whether there are any AEs of nocturnal versus morning dosing.
